# Rapid Testing of Gene-Gene Interactions in Genome-Wide Association Studies of Binary and Quantitative Phenotypes

**DOI:** 10.1002/gepi.20629

**Published:** 2011-09-21

**Authors:** Kanishka Bhattacharya, Mark I McCarthy, Andrew P Morris

**Affiliations:** 1 Wellcome Trust Centre for Human Genetics, University of OxfordOxford, United Kingdom; 2 Oxford Centre for Diabetes, Endocrinology and Metabolism, University of OxfordOxford, United Kingdom

**Keywords:** genome-wide association study, gene-gene interaction, computational efficiency

## Abstract

Genome-wide association (GWA) studies have been extremely successful in identifying novel loci contributing effects to a wide range of complex human traits. However, despite this success, the joint marginal effects of these loci account for only a small proportion of the heritability of these traits. Interactions between variants in different loci are not typically modelled in traditional GWA analysis, but may account for some of the missing heritability in humans, as they do in other model organisms. One of the key challenges in performing gene-gene interaction studies is the computational burden of the analysis. We propose a two-stage interaction analysis strategy to address this challenge in the context of both quantitative traits and dichotomous phenotypes. We have performed simulations to demonstrate only a negligible loss in power of this two-stage strategy, while minimizing the computational burden. Application of this interaction strategy to GWA studies of T2D and obesity highlights potential novel signals of association, which warrant follow-up in larger cohorts. *Genet. Epidemiol*. 2011.© 2011 Wiley Periodicals, Inc.35: 800-808, 2011

## INTRODUCTION

In the search for novel loci contributing effects to complex human traits, the success of genome-wide association (GWA) studies has been well published [McCarthy et al., 2008]. These studies have improved our understanding of the genetic architecture of complex traits, often implicating pathways that might previously have been overlooked as promising biological candidates. However, despite this success, much of the genetic component of most complex traits is still, as yet, unexplained. For example, despite meta-analysis of GWA studies of many thousands of individuals from closely related populations, not more than 10% of the familial aggregation of type 2 diabetes (T2D) can be attributed to more than 35 established disease loci [Dupuis et al., 2010; Voight et al., 2010].

GWA studies are typically analyzed using single-locus methods, testing for the association of the trait with each SNP, in turn, across the genome. This approach is well powered to detect association with common causal variants with moderate marginal effects on the trait [The Wellcome Trust Case Control Consortium, 2007]. However, there is increasing evidence from model organisms that quantitative traits may be influenced by complex interplay between genes [Flint and MacKay, 2009], with the consequence that the effect of genotypes at one locus is modified, or even masked, by genotypes at other loci [Cordell, 2009]. Within this paradigm, individual causal variants need not exhibit strong marginal effects, but together may contribute to the overall trait variance. If these gene-by-gene (G × G) interactions also exist in humans, they may thus account for some of the “missing heritability” of complex traits, but will not be easily identified through single-SNP analysis of GWA studies, irrespective of sample size.

To date, there have been few examples of significant evidence of G × G interaction in human GWA studies. Recently, “compelling” evidence of interaction between SNPs in *HLA-C* and *ERAP1* has been demonstrated in a GWAS of psoriasis, although both these loci demonstrate strong marginal effects on the disease [The Genetic Analysis of Psoriasis Consortium and the Wellcome Trust Case Control Consortium 2, 2010]. The fact that very few interactions have been observed may not preclude their existence. Rather, this may simply reflect the reluctance of researchers to undertake GWA interaction studies, primarily because of the large number of tests that are required. For example, in a GWA study of 500,000 SNPs, a complete two-locus interaction scan of the genome necessitates ^500,000^C_2_ (500,000 “choose” 2) or ∼125 billion tests. As a result, a more stringent significance threshold is required for G × G interactions than for marginal effects in order to allow for the burden of additional multiple testing. Alternative strategies have been proposed to reduce the number of tests required by focusing, for example, only on interactions between variants with at least some marginal evidence of association [Evans et al., 2006]. However, over a wide range of models of G × G interactions, such two-stage strategies lack power compared to a full two-locus scan of the genome, despite the reduced penalty for multiple testing. With such stringent significance levels, much larger sample sizes will be required to detect interaction effects with the same contribution to the phenotypic variance than marginal effects identified through single-locus analysis. Nevertheless, meta-analysis of GWA studies across large-scale international consortia [Barrett et al., 2009; Dupuis et al., 2010; Voight et al., 2010; Lango Allen et al., 2010] may provide sufficient power to detect large G × G interaction effects, provided that an appropriate statistical testing framework is available.

An additional challenge arising from the number of tests in GWA G × G interaction studies is computational, particularly with the large sample sizes that will be required to detect these effects. We typically assess the statistical evidence for interaction effects by means of likelihood-ratio tests in a generalized linear modeling (GLM) framework, which can accommodate both quantitative and dichotomous traits. This framework is extremely flexible and can allow for covariates to adjust for potential environmental risk factors or population structure. However, maximum-likelihood solutions for these models generally require application of numerical optimization algorithms, which are computationally demanding, and thus infeasible for GWA studies using a single processor. To alleviate this problem, Purcell et al. [2007] have implemented a “fast-epistasis” procedure in PLINK to allow rapid-testing of G × G interactions for dichotomous traits. Their approach focuses on a comparison of odds ratios from a 2 × 2 contingency table of allele counts at a pair of SNPs between cases and controls (Table I). They demonstrate that *P*-values from the “fast-epistasis” procedure approximate those from a GLM, and thus can be used for screening of pairs of SNPs for detailed follow-up with more complex interaction modeling techniques, with minimal computation cost.

**Table I:** Representation of genotype data at two SNPs in case and control cohorts

	SNP 2		
	
SNP 1	MM	Mm	mm
*Cases*			
MM	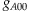	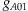	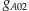
Mm	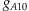	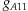	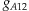
mm	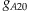	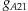	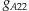
*Controls*			
MM	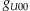	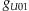	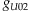
Mm	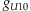	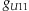	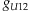
mm	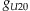	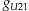	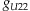

	SNP 2		
		
SNP 1	M	m	
*Cases*			
M	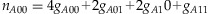	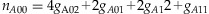	
m	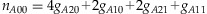	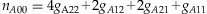	
*Controls*			
M	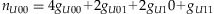	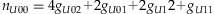	
m	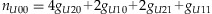	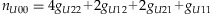	

In this study, we propose a two-stage strategy for rapid testing of G × G interactions in GWA studies of quantitative traits. In the first stage, all pairs of SNPs are screened for evidence of interaction using a computationally efficient test akin to the “fast-epistasis” approach in PLINK. In the next stage, all those pairs of SNPs achieving a nominal significance threshold are carried forward for more detailed modeling in the GLM framework. We perform simulations to determine an appropriate significance threshold for screening interactions in the rapid-testing stage so as to minimize computation time without substantial loss of power compared to a complete two-locus scan of the genome in the GLM framework. Our testing strategy has been applied to GWA G × G interaction studies of T2D and obesity [The Wellcome Trust Case Control Consortium, 2007]. Our results highlight potential novel signals of association that would not have been identified through traditional single-locus analysis, but which warrant follow-up in larger cohorts.

## MODEL AND METHODS

### MODEL FORMULATION AND ANALYSIS FRAMEWORK

Consider a sample of *N* unrelated individuals genotyped for two SNPs. We denote the genotypes of the *i*th individual at these two SNPs by 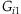 and 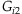, respectively. Genotypes are coded as 0 for the common homozygote, 1 for the heterozygote and 2 for the rare homozygote. Here, we consider binary or quantitative phenotypes, denoted *y*_*i*_ for the *i*th individual. Then, under the assumption of an additive main effect of each SNP on the phenotype, β_1_ and β_2_, and an additive-additive interaction effect, β_12_, it follows that


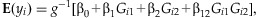
(1)

where *g* is the link function in a GLM framework. Within this framework, we can construct a likelihood ratio test of interaction between the two SNPs, Λ, by comparing the deviance of model (1) when β_12_ = 0, with that when β_12_ is unconstrained. The precise form of the test depends on the phenotype under investigation (Appendix), and has an approximate χ^2^ distribution with one degree of freedom, with a resulting *P*-value denoted *P*_GLM_.

The GLM framework is extremely flexible, and can be adapted to incorporate covariates to allow for nongenetic risk factors or population structure. However, obtaining model parameter estimates and testing for interactions between pairs of SNPs requires numerical algorithms that are too computationally demanding to be applied on a genome-wide scale.

### RAPID G × G INTERACTION TESTING: BINARY PHENOTYPES

In the context of a binary phenotype, a computationally efficient approach for detecting G × G interactions is to compare SNP-SNP association between cases and controls. We begin by constructing contingency tables of alleles at the two SNPs in each phenotype group by collapsing the sample genotype data (Table I). A test of interaction between the SNPs is then given by comparing allelic odds ratios in the two groups, given by


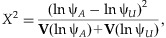


and has an approximate χ^2^ distribution with one degree of freedom, with the resulting *P*-value denoted *P*_FAST_. In this expression,


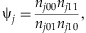


and


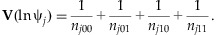


This test has been implemented in PLINK [Purcell et al., 2007], and is invoked using the “fast-epistasis” command. The test statistic, *X*^2^, has a closed form, and thus can be applied to G × G interactions on a genome-wide scale. However, by collapsing genotype data to a contingency table of alleles, we are implicitly assuming Hardy-Weinberg equilibrium at each SNP, and may inflate type I error rates if violated. It is therefore recommended that *X*^2^ is used as a screening tool. All pairs of SNPs passing a pre-determined significance threshold, *P*_FAST_<α_FAST_, are subsequently tested for interaction in the GLM, which is robust to Hardy-Weinberg disequilibrium. The choice of significance threshold represents a trade off between power to detect interaction effects and computation time: the more stringent α_FAST_, the fewer pairs of SNPs are tested in the GLM, but consequently the more likely a true interaction effect will be overlooked.

### RAPID G × G INTERACTION TESTING: QUANTITATIVE PHENOTYPES

One simple approach to construct a rapid test of interaction between pairs of SNPs in the context of a quantitative trait is to dichotomize the phenotype, and proceed as described above. Some study designs focus on the ascertainment of individuals from the extremes of the trait distribution, and thus naturally form a dichotomy. However, when individuals are selected entirely at random with respect to the quantitative trait, we can define a quasi-case-control phenotype, denoted *y*
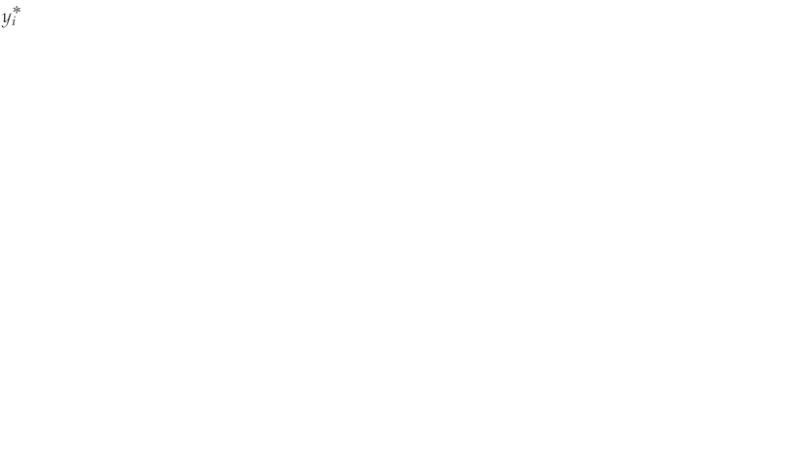
 for the *i*th individual, and given by


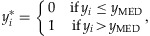


where *y*_MED_ is the median of the distribution.

### INTERACTION TESTING BETWEEN IMPUTED VARIANTS

The methodology described above can be easily applied to imputed genotypes at variants not typed directly as part of the study, but which are present on high-density reference panels, such as those made available through the international HapMap project [The International HapMap Consortium, 2007]. Typically, imputation generates a distribution of possible genotype calls for the *i*th individual at the *j*th SNP, denoted 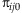, 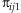 and 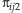, respectively, for the common homozygote, heterozygote and rare homozygote [Marchini et al., 2007; Howie et al., 2009]. In the GLM, we replace the observed genotypes, 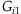 and 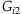, at directly typed SNPs, with their expectations under an allele-dose model, given by


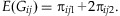


In the rapid G × G interaction test, we replace the contingency table of observed genotype counts with expected counts from the imputation distribution, assuming no LD between the pair of SNPs.

### ADJUSTMENT FOR COVARIATES

The GLM framework is extremely flexible and can be adapted to incorporate covariates to allow for non-genetic risk factors or population structure. However, it is not possible to adjust for these covariates directly in the rapid G × G interaction test of binary or quantitative phenotypes outlined above, without a loss in computational efficiency. Clearly, allowing for covariates only in the GLM in the second stage of analysis may lead to misleading results. We thus recommend the use of residuals after adjustment of the phenotype for covariates in both the initial rapid G × G interaction test and the subsequent detailed modeling analysis in the GLM framework.

### SOFTWARE

The methodology described above has been implemented, for both quantitative and binary phenotypes, in the open-source IntRapid software, and is freely available for download from the website http://www.well.ox.ac.uk/INTRAPID. The software allows specification of the significance threshold, α_FAST_, for carrying forward pairs of SNPs from the rapid interaction screening phase, and can be applied to both directly genotyped and imputed variants.

### SIMULATION STUDY

We have undertaken simulations to evaluate the power of the proposed rapid G × G interaction test in the context of a quantitative trait compared with that of the more computationally intensive GLM. We consider a range of models of two-locus interaction: additive-additive, pure additive, dominant-dominant and recessive-recessive. Each model is parameterized in terms of causal allele frequency at two SNPs, denoted *q*_1_ and *q*_2_, and an interaction component, ε. For any given model, this component can be used to obtain population mean trait values, γ_*jk*_, for each two-locus genotype (Table II).

**Table II:** Two-locus association models incorporating interaction effects utilised in simulations. All models are parameterised in terms of the interaction component ε

	SNP 2		
	
SNP 1	MM	Mm	mm
*Additive-additive interaction*			
MM	γ_00_ = ε–1	γ_01_ = −0.5	γ_02_ = −ε
Mm	γ_10_ = −0.5	γ_11_ = 0	γ_12_ = 0.5
mm	γ_20_ = −ε	γ_21_ = 0.5	γ_22_ = 1 + ε
*Pure additive interaction*			
MM	γ_00_ = ε	γ_01_ = 0	γ_02_ = −ε
Mm	γ_10_ = 0	γ_11_ = 0	γ_12_ = 0
mm	γ_20_ = −ε	γ_21_ = 0	γ_22_ = ε
*Dominant-dominant interaction*			
MM	γ_00_ = 0	γ_01_ = 0	γ_02_ = 0
Mm	γ_10_ = 0	γ_11_ = ε	γ_12_ = ε
mm	γ_20_ = 0	γ_21_ = ε	γ_22_ = ε
*Recessive-recessive interaction*			
MM	γ_00_ = 0	γ_01_ = 0	γ_02_ = 0
Mm	γ_10_ = 0	γ_11_ = 0	γ_12_ = 0
mm	γ_20_ = 0	γ_21_ = 0	γ_22_ = ε

For each model, we generate 1,000 replicates of a sample of 5,000 unrelated individuals. We generate the genotype of each individual at the two SNPs, denoted 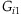 and 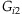, according to the causal allele frequencies, assuming HWE at both variants. The phenotype of the individual is then generated from a Gaussian distribution with mean 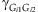 and unit variance. For each replicate of phenotype-genotype data, we perform: (i) the rapid G × G interaction test to obtain *P*_FAST_; and (ii) the GLM interaction test to obtain *P*_GLM_. We then consider a range of significance thresholds, α_FAST_, for carrying forward pairs of SNPs from the rapid interaction screening phase. In particular, we consider α_FAST_ = 1 as our benchmark, since this corresponds to no initial screening step (all pairs of SNPs will be carried forward for testing in the GLM). For each significance threshold, power is estimated by the proportion of replicates for which *P*_FAST_<α_FAST_ and *P*_GLM_ meets a nominal pair-wise genome-wide significance threshold of 10^−10^.

### APPLICATION TO GWA STUDIES OF T2D AND OBESITY

The T2D component of the WTCCC [The Wellcome Trust Case Control Consortium, 2007] consists of 1,999 cases from the Diabetes UK Warren 2 repository, and 3,004 population controls from the 1958 British Birth Cohort (58C) and the UK National Blood Service (NBS). Samples were genotyped using the Affymetrix GeneChip 500K Mapping Array Set that incorporates 500,568 SNPs, genome-wide. The T2D cases were also measured for body mass index (BMI) to assess overall obesity, a phenotype that is typically adjusted for age and sex in downstream analyses.

We utilized the same quality control (QC) filters employed by the WTCCC to exclude samples and SNPs from the analysis, full details of which are presented in the description of the experiment [The Wellcome Trust Case Control Consortium, 2007]. In brief, samples were excluded on the basis of low call rate, outlying genome-wide heterozygosity, discrepancies in WTCCC and external identifying information, non-European ancestry, duplication and apparent relatedness. SNPs were excluded on the basis of low call rate, extreme deviation from HWE, differential allele or genotype frequencies between the two control cohorts, and manual visual inspection of genotype calls in cluster plots. To avoid any problems of sparsity in the two-locus genotype contingency tables (Table I), we restricted our analysis to SNPs with MAF of at least 5%.

For each pair of autosomal SNPs passing QC filters, we tested for: (i) interaction with T2D in WTCCC cases and controls using the rapid approach for binary traits; and (ii) interaction with log_10_BMI residuals after adjustment for age and sex in T2D cases using the rapid approach for quantitative traits. All pairs of SNPs achieving a screening significance threshold of *P*_FAST_<10^−4^ were then tested for interaction in the GLM.

## RESULTS

### SIMULATION STUDY

**Fig. 1:**
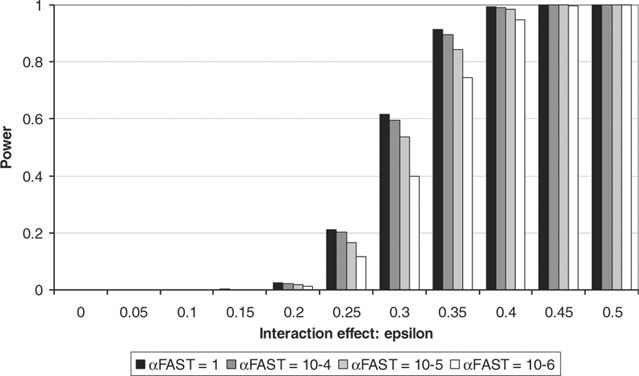
Power, at a nominal significance threshold of *P*_GLM_<10^−10^, of the rapid interaction testing strategy for an additive-additive two-locus model of association, with causal allele frequency of 20% at both SNPs. Power is presented as a function of the additive-additive interaction component, ε (Table II), for a range of thresholds, α_FAST_, for carrying forward pairs of SNPs from the rapid testing stage to the GLM analysis framework.

Figure 1 presents the power, at a nominal significance threshold of *P*_GLM_<10^−10^, of the proposed rapid interaction testing strategy for an additive-additive two-locus model of association, with causal allele frequency of 20% at both SNPs. Power is presented as a function of the additive-additive interaction component, ε (Table II), for a range of thresholds, α_FAST_, for carrying forward pairs of SNPs from the rapid testing stage. There is only a minimal loss in power for a threshold of α_FAST_ = 10^−4^, compared to the benchmark of α_FAST_ = 1 where all pairs of SNPs are tested for interaction in the computationally intensive GLM framework. However, for more stringent thresholds, the reduction in power is more noticeable.

**Fig. 2:**
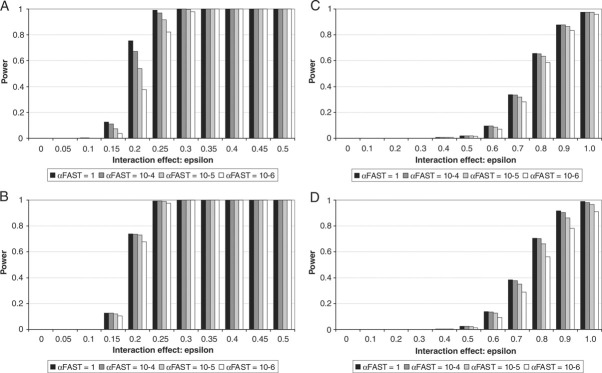
Power, at a nominal significance threshold of *P*_GLM_<10^−10^, of the rapid interaction testing strategy for two-locus models of association with causal allele frequency of 50% at both SNPs: (**A**) additive-additive; (**B**) pure additive; (**C**) dominant-dominant; and (**D**) recessive-recessive. Power is presented as a function of the additive-additive interaction component, **ε** (Table II), for a range of thresholds, α_FAST_, for carrying forward pairs of SNPs from the rapid testing stage to the GLM analysis framework.

Figure 2 presents the power, at a nominal significance threshold of *P*_GLM_<10^−10^, of the proposed rapid interaction testing strategy for two-locus models of association with causal allele frequency of 50% at both SNPs: (A) additive-additive; (B) pure additive; (C) dominant-dominant; and (D) recessive-recessive. Power is presented as a function of the additive-additive interaction component, ε (Table II), for a range of thresholds, α_FAST_, for carrying forward pairs of SNPs from the rapid testing stage. As before, there is only a minimal loss in power for a threshold of α_FAST_ = 10^−4^, compared to the benchmark of α_FAST_ = 1. The conclusions are consistent across the range of interaction models considered, despite the fact that our analyses assume additive effects only.

### APPLICATION TO GWA STUDY OF T2D

A total of 4,862 individuals and 357,775 SNPs with MAF >5% passed QC filters. Using a rapid interaction testing threshold of α_FAST_ = 10^−4^, a total of 6.17 million pairs of SNPs were carried forward for consideration in the more computationally intensive GLM framework. This analysis took 312 hr of computing time on a dedicated processor, compared to an expected 4,389 hr to test for interaction between all pairs of SNPs in the GLM framework.

No pairs of SNPs met a Bonferroni correction for multiple testing of all pairs of SNPs passing QC filters (*P*<7.8 × 10^–13^). Table III presents lead SNPs at each pair of loci demonstrating strong evidence for interaction (*P*<10^−10^) in the second-stage GLM, together with *P*-values from the first stage rapid test. Also presented are *P*-values for each SNP from a marginal single-locus test of association from a GLM incorporating only an additive effect. None of these SNPs show evidence of marginal association with T2D, and thus would not have been discovered through single-locus GWAS analysis under the assumption of an additive effect in the log-odds ratio.

**Table III:** Lead SNPs at pairs of loci with strong evidence of interaction (*P*<10^−10^) in a GWA study of T2D

SNP 1					SNP 2					Interaction *p*-value		Single-locus *p*-value	
			
ID	Chromosome	Position	Locus	MAF	ID	Chromosome	Position	Locus	MAF	Rapid	GLM	SNP1	SNP2
rs6421008	8	135581827	*ZFAT*	0.05	rs7827545	8	135635749	*ZFAT*	0.32	1.8 × 10^−14^	1.6 × 10^−11^	5.1 × 10^−1^	1.8 × 10^−3^
rs10916293	1	224738665	*OBSCN*	0.33	rs9314349	8	27530121	*CLU*	0.38	1.5 × 10^−11^	2.8 × 10^−11^	5.7 × 10^−1^	9.0 × 10^−1^

For each SNP, the nearest gene in the locus is indicated. Rapid and GLM interaction *P*-values are obtained from the two stages of the IntRapid analysis. Single-locus *P*-values are obtained from a GLM without interaction effects.

The strongest signal of interaction with T2D was detected between a pair of proximal SNPs in the *ZFAT* gene. The SNPs are only weakly correlated with each other (*r*^2^ = 0.062 and *D*′ = 1.000 in CEU 1000 Genomes Project pilot data), and this interaction could represent a haplotype effect where the risk alleles have a synergistic effect when in *cis*, for example. The *ZFAT* gene encodes a protein that likely functions as a transcriptional regulator involved in apoptosis, but has not been previously implicated in T2D or other metabolic traits. The second strongest signal of interaction with T2D was detected between a pair of SNPs in the *OBSC* gene and flanking the *CLU* gene, respectively. These genes have not been previously implicated in T2D. However, both genes are involved in carbohydrate and lipid metabolic process and in apoptosis, and thus might be reasonable candidates for interaction within related functional pathways.

### APPLICATION TO GWA STUDY OF OBESITY

A total of 1,903 T2D cases and 375,159 SNPs with MAF>5% passed QC filters. We considered residuals of log_10_BMI, after adjustment for age and sex, as our phenotype. Using a rapid interaction testing threshold of α_FAST_ = 10^−4^, a total of 7.14 million pairs of SNPs were carried forward for consideration in the more computationally intensive GLM framework. This analysis took 127 hr of computing time on a dedicated processor, compared to an expected 4,826 hr to test for the interaction between all pairs of SNPs in the GLM framework.

No pairs of SNPs met a Bonferroni correction for multiple testing of all pairs of SNPs passing QC filters (*P*<7.1 × 10^−13^). Table IV presents lead SNPs at each pair of loci demonstrating strong evidence for interaction (*P*<10^−10^) in the second-stage GLM, together with *P*-values from the first-stage rapid test. Also presented are *P*-values for each SNP from a marginal single-locus test of association from a GLM incorporating only an additive main effect. None of these SNPs show evidence of marginal association with BMI (adjusted for age and sex), and thus would not have been discovered through single-locus GWAS analysis under the assumption of an additive effect in the log-odds ratio. The loci implicated in the four strongest signals of pair-wise interaction have not been previously implicated in obesity or other metabolic traits.

**Table IV:** Lead SNPs at pairs of loci with strong evidence of interaction (*P*<10^−10^) in a GWA study of BMI adjusted for age and sex

SNP 1					SNP 2					Interaction *P*-value		Single-locus *P*-value	
			
ID	Chromosome	Position	Locus	MAF	ID	Chromosome	Position	Locus	MAF	Rapid	GLM	SNP1	SNP2
rs9460779	6	22855116	*HDGFL1*	0.13	rs9928199	16	75649112	*MON1B*	0.09	1.2 × 10^−5^	2.5 × 10^−11^	9.9 × 10^−1^	1.5 × 10^−2^
rs12655480	5	178212877	*ZNF354B*	0.35	rs2423646	20	12212116		0.39	2.3 × 10^−8^	2.5 × 10^−11^	6.9 × 10^−1^	2.9 × 10^−1^
rs10067788	5	151313204	*GLRA1*	0.07	rs1023446	16	19321487	*TMC5*	0.37	3.2 × 10^−6^	3.3 × 10^−11^	6.2 × 10^−1^	1.6 × 10^−1^
rs17684830	3	60910010	*FHIT*	0.17	rs521446	9	133647868	*VAV2*	0.47	1.0 × 10^−6^	5.5 × 10^−11^	7.4 × 10^−1^	4.7 × 10^−1^

For each SNP, the nearest gene in the locus is indicated. Rapid and GLM interaction *P*-values are obtained from the two stages of the IntRapid analysis. Single-locus *P*-values are obtained from a GLM without interaction effects.

## DISCUSSION

Interactions between variants may contribute to the missing heritability of complex traits. However, such interactions have not typically been studied in GWA analyses because of a fear of a lack of power due to the large number of statistical tests, and the burden of computation. Emily et al. [2009] identified four potential interactions in GWA studies of Crohn's disease, bipolar disease, hypertension and rheumatoid arthritis [The Wellcome Trust Case Control Consortium, 2007], although these signals have yet to be followed-up in independent replication cohorts. More recently, investigation of interaction effects between pairs of SNPs within established loci for psoriasis identified the first G × G interactions with “compelling” evidence of association through GWA studies [The Genetic Analysis Of Psoriasis Consortium and The Wellcome Trust Case Control Consortium 2, 2010].

In this study, we have developed a novel GWA G × G interaction testing scheme, applicable to both binary phenotypes and quantitative traits. We employ a two-stage strategy, implemented in the IntRapid software. In the first stage, all pairs of SNPs are tested in a computationally efficient “rapid” interaction test. For a binary phenotype, our rapid interaction test is equivalent to the “fast-epistasis” approach utilized in PLINK for case-control association analysis [Purcell et al., 2007]. For a quantitative trait, we simply dichotomize the phenotype at the median, creating “pseudo-cases” and “pseudo-controls”, which can then be analyzed in the same way. In the second stage, all pairs of SNPs meeting a pre-determined significance threshold are carried forward for detailed analyses in a more computationally demanding GLM framework.

We have undertaken simulations to evaluate the power of the proposed two-stage rapid G × G interaction test in the context of a quantitative trait compared with that of the more computationally intensive GLM. Over a range of models of two-locus interaction, our results suggest that there is only a minimal loss in power compared to testing all pairs of SNPs in the GLM by carrying forward only those pairs meeting a significance threshold of *P*_FAST_<10^−4^ in the rapid testing stage. In this way, the number of regressions performed in the computationally intensive GLM framework is minimized, substantially reducing computation time, but at only minimal cost in terms of reduced power.

We have applied IntRapid to GWA studies of T2D and obesity [The Wellcome Trust Case Control Consortium, 2007] using a rapid-interaction testing significance threshold of *P*_FAST_<10^−4^. The rapid testing strategy reduced computation time by an order of magnitude compared with a full two-locus scan of the genome in a GLM framework. The rapid-interaction testing stage can also easily be parallelized, further reducing computation if a cluster of processors is available for analysis. Although our analysis did not reveal genome-wide significant evidence of interaction (at a Bonferroni corrected threshold), our results highlighted two pairs of loci with strong evidence of interaction (*P*<10^−10^) with T2D and four pairs of loci with strong evidence of interaction with BMI (adjusted for age and sex). Our T2D analysis highlighted an interaction between a pair of weakly correlated SNPs in the *ZFAT* gene, and an interaction between SNPs in genes involved in metabolic process and apoptosis. The biological relevance of the loci identified in the obesity interaction analysis is not so obvious. Further investigation of the potential interactions between pathways in which all these genes act is necessary. Although interesting, caution is advised to avoid over-interpretation of these results, particularly given the relatively small sample size of the GWA study for detecting interaction effects. Follow-up of these results in independent replication cohorts is now required to confirm their relevance to T2D and obesity.

The coming months promise an exciting period of research and development into methodology for the detection of G × G interactions and their application to GWA studies. With ever increasing sample sizes, made possible through meta-analysis of GWA studies across large-scale international consortia, we are perhaps now in a position, for the first time, to detect strong interaction effects for many complex traits, even with the enormous burden of multiple testing. Furthermore, with the availability of computationally efficient software, such as IntRapid, we expect that GWA G × G interaction studies will be a natural addition to traditional single-locus analysis, with the potential to discover many novel loci contributing effects to complex human traits.

## APPENDIX

Within the generalized linear modeling framework, we can construct a likelihood ratio test of interaction between the two SNPs, Λ, by comparing the deviance of model (1) when β_12_ = 0, with that when β_12_ is unconstrained. The precise form of the test depends on the phenotype under investigation, and has an approximate χ^2^ distribution with one degree of freedom.

### BINARY PHENOTYPE

In the context of a binary phenotype, the deviance is given by

In this expression, the log-likelihood

is maximized over all unconstrained parameters, where

### QUANTITATIVE PHENOTYPE

In the context of a quantitative phenotype, the deviance is given by

In this expression, the log-likelihood

is maximized over all unconstrained parameters, where
